# Resting Metabolic Rate of Individuals

**DOI:** 10.3390/metabo13080926

**Published:** 2023-08-08

**Authors:** Abel Plaza-Florido, Juan M. A. Alcantara

**Affiliations:** 1 Pediatric Exercise and Genomics Research Center, Department of Pediatrics, School of Medicine, University of California at Irvine, Irvine, CA 92617, USA; aplazafl@hs.uci.edu; 2 Department of Health Sciences, Institute for Innovation & Sustainable Food Chain Development, Public University of Navarre, 31006 Pamplona, Spain; 3 Navarra Institute for Health Research, IdiSNA, 31008 Pamplona, Spain; 4 Centro de Investigación Biomédica en Red Fisiopatología de la Obesidad y Nutrición (CIBERobn), Instituto de Salud Carlos III, 28029 Madrid, Spain

The resting metabolic rate (RMR) represents the energy required to sustain normal body functions and homeostasis in an awake individual under ambient thermoneutral conditions and during rest. The RMR is the most relevant 24-h energy expenditure (EE) component. In fact, the RMR (i.e., resting EE) commonly accounts for 60–70% of the total 24-h EE, making it a potential indicator of future weight (re)gain. This special issue of *Metabolites*, “Resting Metabolic Rate of Individuals”, contains five original articles and one narrative review, which can be organized into four topics: (i) estimation of RMR using predictive equations; (ii) association between RMR and circulating cardiometabolic risk factors; (iii) effects of two different interventions on RMR; and (iv) RMR in persons with narcolepsy.

Regarding the estimation of RMR, Maury-Sintjago et al. [1] observed, in a sample of 41 Chilean women (age ranged from 18 to 28 yrs.) with overweight or obesity, that different predictive equations overestimated the RMR (resting EE) compared to indirect calorimetry, which is the gold standard technique for assessing the RMR [2,3,4]. Briefly, in women with overweight, they observed that FAO/WHO/UNU (1985), FAO/WHO/UNU (2004), Harris–Benedict, and Mifflin–St Jeor equations overestimated the RMR. Similar results were observed for women with obesity [1]. Nevertheless, the Mifflin–St Jeor predictive equation showed the lowest overestimation of RMR in both weight status groups; thus, it is the equation recommended by the authors for this population [1].

Two cross-sectional studies reported the associations between RMR and cardiometabolic risk factors in middle-aged [5] and young adults [6], respectively. Soares et al. [5] reported that middle-aged adults (aged: 41 ± 15 yrs.; 67% female) without metabolic syndrome (n = 95) presented lower RMR values (1432 ± 21 vs. 1376 ± 21 kilocalories per day (kcal/day)) compared to middle-aged adults with metabolic syndrome (n = 85; aged: 55 ± 11 yrs.; 52% female). These between-group differences remained statistically significant after adjusting for covariates such as age, sex, ethnicity, and body composition, among others [5]. Interestingly, they observed that each increase in the number of metabolic syndrome components was positively associated with RMR values [5]; in other words, the higher the number of metabolic syndrome components, the higher the RMR. In another study, Alcantara et al. [6] observed that young men (n = 35; aged: 23 ± 2 yrs.) yielded higher intra-assessment RMR variability values (determined using the coefficient of variation [CV]; expressed in %) compared to young women (n = 72; aged: 22 ± 2 yrs.). Specifically, higher CVs for resting volume of oxygen consumption (adjusted mean difference of 6 ± 2%), volume of carbon dioxide production (adjusted mean difference of 5 ± 1%), and EE (adjusted mean difference of 3 ± 1%) were observed in men compared to women [6]. Likewise, an inverse association was reported between the intra-assessment RMR variability and vagal-related heart rate variability parameters in men (standardized β coefficients ranged from −0.36 to −0.38), but not in women [6].

Two other studies were published which investigated the effects of exercise (longitudinal effects) [7] and of whole-body electrical stimulation (acute effects) [8] on resting EE. Taousani et al. [7] enrolled 48 women with gestational diabetes mellitus (aged <40 yrs.), and 43 completed the intervention. Women were allocated into three different groups: (i) usual care (n = 17); (ii) walking (n = 14); and (iii) combined aerobic and strength exercises (mixed exercise group; n = 12). Detailed information regarding the training sessions can be found elsewhere [7]. Briefly, exercise sessions were performed between the 27th and 38th gestational weeks, and both exercise modalities similarly increased RMR after the intervention compared to the usual care group (post-intervention minus pre-intervention differences were 31, 243, and 264 kcal/day for usual care, walking, and the mixed exercise groups, respectively) [7]. Interestingly, the intervention did not appear to influence resting carbohydrate utilization [7]. In the study conducted by Perez de Arrilucea Le Floc’h et al. [8], the effects of different electrical frequencies of whole-body electrical stimulation (1 hertz [Hz], 2 Hz, 4 Hz, 6 Hz, 8 Hz, and 10 Hz) on resting EE, and on EE during uphill walking, were examined in a sample of 10 healthy young men (aged 22 ± 3 yrs.). At rest, they observed that 4 Hz had the largest impact on resting EE, increasing the EE by >600% (stimulated condition vs. unstimulated condition: Δ = 8.9 ± 1.5 kcal/min) [8]. The same impact as that observed for EE was noticed for the respiratory exchange ratio, as a remarkable increase in this parameter was observed [8]. Further information regarding the uphill walking condition (e.g., methodology, results) can be found elsewhere [8], as this Editorial is focused on resting EE.

Lastly, a review article was included in this Special Issue. Dhafar et al. [9] conducted a narrative review regarding narcolepsy and its relationship with changes in body weight and RMR. In addition, they extensively discussed potential mechanisms of weight gain and metabolic changes and proposed future research agendas on this topic. In summary, the authors concluded that based on the current literature, no relevant changes in RMR have been observed in patients with narcolepsy compared to control individuals [9].

The present Special Issue summarizes the progress on the topic of RMR and human health in different populations, which will be of interest from both clinical and research perspectives. In addition, it highlights the current limitations of certain research areas and the need for more studies to be carried out in order to advance scientific knowledge.
